# Do highly divergent loci reside in genomic regions affecting reproductive isolation? A test using next-generation sequence data in *Timema* stick insects

**DOI:** 10.1186/1471-2148-12-164

**Published:** 2012-08-31

**Authors:** Patrik Nosil, Thomas L Parchman, Jeffrey L Feder, Zach Gompert

**Affiliations:** 1 Department of Ecology and Evolutionary Biology, University of Colorado, Boulder, 80303, USA; 2 Department of Botany, University of Wyoming, Laramie, WY, 82071, USA; 3 Department of Biology, Notre Dame University, South Bend, IN, 11111, USA

**Keywords:** Ecological speciation, Gene flow, Genomic cline, Genomic islands of speciation, Next-generation sequencing, Reproductive isolation

## Abstract

**Background:**

Genetic divergence during speciation with gene flow is heterogeneous across the genome, with some regions exhibiting stronger differentiation than others. Exceptionally differentiated regions are often assumed to experience reduced introgression, i.e., reduced flow of alleles from one population into another because such regions are affected by divergent selection or cause reproductive isolation. In contrast, the remainder of the genome can be homogenized by high introgression. Although many studies have documented variation across the genome in genetic differentiation, there are few tests of this hypothesis that explicitly quantify introgression. Here, we provide such a test using 38,304 SNPs in populations of *Timema cristinae* stick insects. We quantify whether loci that are highly divergent between geographically separated (‘allopatric’) populations exhibit unusual patterns of introgression in admixed populations. To the extent this is true, highly divergent loci between allopatric populations contribute to reproductive isolation in admixed populations.

**Results:**

As predicted, we find a substantial association between locus-specific divergence between allopatric populations and locus-specific introgression in admixed populations. However, many loci depart from this relationship, sometimes strongly so. We also report evidence for selection against foreign alleles due to local adaptation.

**Conclusions:**

Loci that are strongly differentiated between allopatric populations sometimes contribute to reproductive isolation in admixed populations. However, geographic variation in selection and local adaptation, in aspects of genetic architecture (such as organization of genes, recombination rate variation, number and effect size of variants contributing to adaptation, etc.), and in stochastic evolutionary processes such as drift can cause strong differentiation of loci that do not always contribute to reproductive isolation. The results have implications for the theory of ‘genomic islands of speciation’.

## Background

Levels of genetic differentiation are highly variable across the genome [1-5]. In particular, regions of the genome harboring genes under divergent selection or causing reproductive isolation might exhibit accentuated differentiation between populations, for example because such regions are resistant to introgression. In this context, it is important to realize that divergent selection itself can generate reproductive isolation, for example via ecologically-based selection against immigrants and hybrids [6-10]. Reproductive isolation can also arise due to negative interactions between loci that cause intrinsic post-zygotic isolation (i.e., Dobzhansky-Muller incompatibilities, DMIs), and DMIs can evolve by selection or drift [11-15]. Introgression can be less impeded at neutrally evolving regions or those not involved in DMIs, leading to low differentiation. Thus, genomic divergence may be particularly heterogeneous during the process of population divergence with gene flow, during which genetic differentiation accumulates in some regions, while the homogenizing effects of introgression prevent differentiation of other regions [1,5]. Such ideas concerning heterogeneous genomic divergence have a long history in the hybrid zone literature [16-18], but have been revitalized by new genomic studies [19-28].

But many processes other than selection and reproductive isolation can promote genetic differentiation. If overall gene flow levels are very low, for example due to geographic isolation, then pronounced genetic differentiation might arise due to the stochastic effects of genetic drift [29-31]. These stochastic effects might be accentuated by variable mutation rates and by low levels of recombination [30-32]. These points have led some to question whether highly differentiated regions necessarily harbor genes affecting reproductive isolation [32,33]. Likewise, recent divergence and inadequate time for lineage sorting might result in weak differentiation of regions that will eventually become highly differentiated [32,33]. Finally, geographic variation in aspects of genetic architecture such as recombination rate variation (including chromosomal inversion polymorphism), and in local selective regimes, could result in different genomic regions being differentiated or resistant to introgression in different parts of a species range [34-37]. Collectively, these issues raise the question of the extent to which highly divergent genomic are causally important for speciation versus being incidental consequences of divergence between populations already undergoing little or no introgression [33].

There are several ways to test if highly differentiated regions reside in regions of reduced introgression. First, mapping studies could test whether phenotypic traits involved in divergent adaptation or causing reproductive isolation map to regions of strong divergence between natural populations [38,39]. Second, experiments might measure selection on the genome directly [40-42], and ask if divergent selection acts on regions of strong differentiation. Third, one might estimate if genomic regions that are exceptionally differentiated between allopatric populations undergo atypical patterns of introgression in zones of admixture [20,43].

Here, we adopt this third approach following [43] using a previously published dataset from *Timema cristinae* stick insects. The genotyping-by-sequencing approach which generated these data used restriction enzymes to cut up the genome into DNA fragments that are distributed across the genome, sequenced tens of millions of these fragments on the Illumina next-generation sequencing platform, and aligned the fragments to discover genetic variation (facilitated by each specimen being individually barcoded). This approach was thus aimed at surveying genome wide patterns of differentiation, rather than focusing in on specific genes that causally affect adaptation and speciation. Previous work used this dataset to test which ecological and geographic factors affect variability in genetic differentiation (i.e., in F_ST_) [44]. The results revealed that the number of exceptionally differentiated ‘outlier loci’, allele-frequency clines, and the overall distribution of genomic differentiation were recognizably affected by multiple factors such as host-plant use, geographic distance, climatic variability, and selection to avoid maladaptive hybridization (i.e., reinforcement) [44]. Previous work did not estimate variation across the genome (i.e., across loci) in levels of introgression. Thus, the novel prediction we test here is that there will be an association between locus-specific divergence and locus-specific introgression, consistent with highly differentiated loci contributing to reproductive isolation.

**Figure 1:**
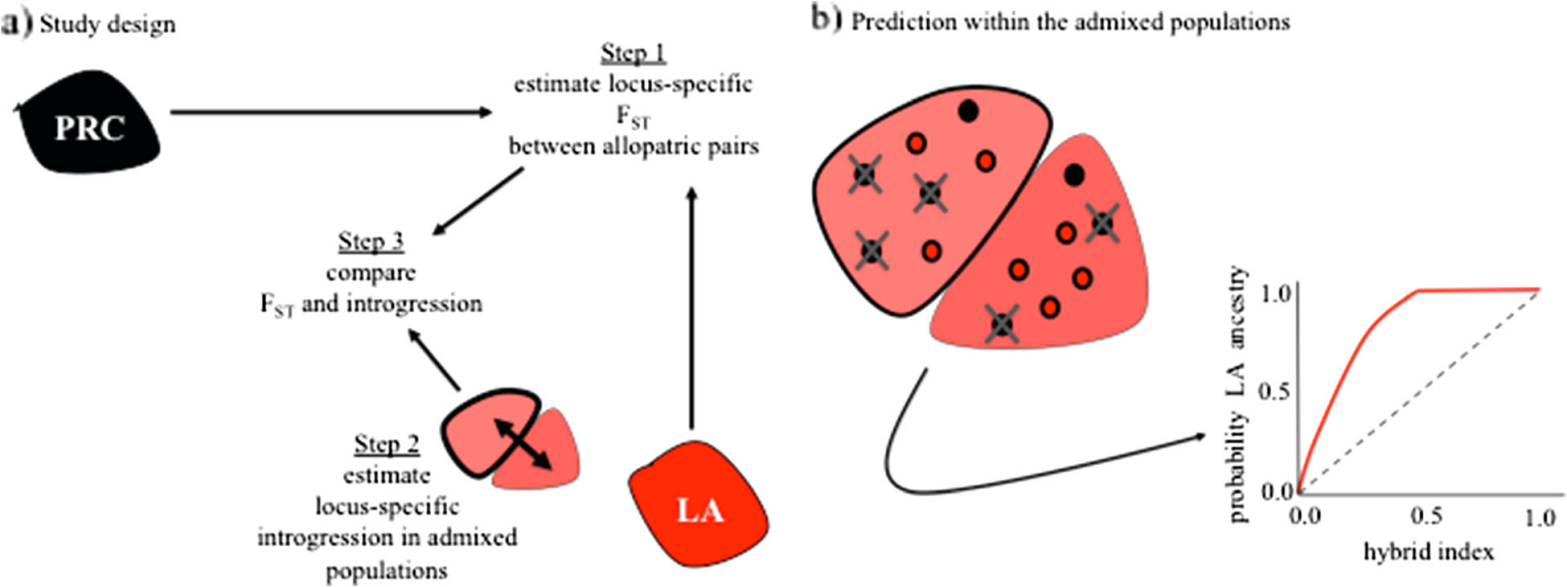
**Description of the study design and associated predictions.a**) The design involves three steps. First, locus-specific genetic divergence is estimated between the allopatric populations LA and PRC. Second, locus-specific introgression is estimated in two admixed populations (HV and MR). Third, the results from the first two steps are compared. **b**) The two admixed populations examined are geographically closer to LA than PRC (Additional file 1: Figure S1). Thus, the environmental conditions in the admixed populations might mirror those in LA, resulting in selection against alleles from PRC (denoted by ‘X’s representing selection against black alleles). This process could result in excess LA ancestry for a given value of hybrid index, as depicted in the genomic cline figure (bottom right).

Our approach involves three steps (Figure 1). First, we estimate locus-specific genetic divergence (i.e., F_ST_) between two allopatric (i.e., non-admixed) populations of *T. cristinae*. This step included, but was not restricted to, the identification of high-F_ST_ outlier loci (which can evolve by selection or drift, but tend to be enriched for those experiencing selection) [44]. Second, for the same loci used in step one, we estimate patterns of locus-specific introgression in admixed populations. Third, we quantify the correspondence between locus-specific divergence for allopatric populations and locus-specific introgression in admixed populations. We find substantial, but far from extreme, correspondence between divergence and introgression. The previous study using this dataset also reported evidence for local adaptation to climatic conditions, by virtue of documenting associations between allele frequencies at specific loci and climatic variables [44]. However, past work did not test for selection against foreign alleles, as would be predicted by local adaptation. We expand past work by reporting evidence for selection against foreign alleles in the admixed populations due to local adaptation. The results shed insight into the relationship between genomic divergence and introgression during speciation. 

### The study system

*Timema* are wingless, herbivorous insects inhabiting Southwestern North America [45]. The current study considers *T. cristinae*, which uses two host species (*Adenostoma fasciculatum*: Rosaceae and *Ceanothus spinosus*: Rhamnaceae) [46]. Populations using different hosts exhibit heritable differences in a suite of characteristics, such as color, color pattern, body size, and body shape. These differences generate extrinsic reproductive isolation due to ecologically based selection against between-host migrants and hybrids, and have also driven the evolution of sexual isolation due to reinforcement. In contrast to this extrinsic and sexual isolation, DMIs appear absent in this system [46].

Host patches used by *T. cristinae* are sometimes separated from patches of the alternative host, usually via regions containing unsuitable hosts. All populations found in such geographically separated patches are termed ‘allopatric’. We study here two allopatric populations, LA and PRC, which are known from past work to be strongly genetically differentiated, undergo little or no between-host gene flow, and are thus ‘non-admixed’ [44,47-50]. Our estimates of genetic divergence (i.e., F_ST_) here thus all stem from LA and PRC (Figure 1).

In contrast to allopatric scenarios, the two host species are sometimes distributed in adjacent patches that are in direct geographic contact with one another. Previous work has shown that such adjacent populations undergo extensive between-host gene flow and are ‘admixed’ [44,47-50]. We study two such admixed populations here, HV and MR, which exhibit very weak genetic divergence and high gene flow between hosts [44,47-50]. For the SNP data analyzed here estimates of Burrow’s composite measure of Hardy Weinberg and linkage disequilibrium (Δ) within populations [51] were very low, indicating the SNPs were largely statistically independent from one another (e.g., mean Δ across locus pairs was as follows: HVA, 0.0038; HVC, 0.0035; LA, 0.0030; MR1A, 0.0040; MR1C, 0.0034; PRC, 00026; with ‘A’ designating use of *Adenostoma* as a host and ‘C’ use of *Ceanothus*) [44]. Nonetheless, as expected if HV and MR were admixed, estimates of Δ within these populations were significantly greater than those for LA and PRC (F_1,6_ = 13.76, p = 0.021). Our estimates of introgression here thus all stem from the admixed populations HV and MR.

### The genomic clines approach

The analytical approach we use is that of ‘genomic clines’ [52]. Genomic clines are mathematical functions that describe the probability of locus-specific ancestry along a gradient in genome-wide admixture or hybrid index. Hybrid index (h) is defined as the proportion of an admixed individual’s genome inherited from one of two parental populations (here h = 1 denotes pure LA ancestry and h = 0 denotes pure PRC ancestry).

We thus measured genomic introgression of LA and PRC genetic regions in admixed HV and MR *T. cristinae*. We were not concerned with the geography of introgression, but rather with the movement of genetic material from one genomic background into another within a geographic region i.e., genomic introgression [18,20,52-54]. We quantified locus-specific genomic introgression using the Bayesian genomic cline model on the basis of two locus-specific genomic cline parameters [52]. These cline parameters specify the probability that individual *j* with hybrid index H = h inherited a gene copy at locus I = i from LA (denoted φ; the probability of PRC ancestry is 1 − φ). The base probability of LA ancestry for a locus is equal to an individual’s hybrid index. The genomic cline center parameter, α, specifies an increase (positive values of α) or decrease (negative values of α) in the probability of LA ancestry for a locus relative to the base expectation. The genomic cline rate parameter, β, specifies an increase (positive values) or decrease (negative values) in the rate of transition from low to high probability of LA ancestry as a function of hybrid index and measures the mean ancestry-based pairwise linkage disequilibrium between a locus and all other loci. More formally,

(1) Φ_ij=h_j+2h_j−h_j^2α_i+β_i2h_j−1,

where Φ_ij is given by a simple transformation of Φ_ij to ensure 0≤Φ≤1 and that Φ is a monotonically increasing function of hybrid index. Simulations have demonstrated that selection against specific hybrid genotypes (i.e., locus-specific reproductive isolation), whether arising from single locus (underdominance) or multilocus (Dobzhansky-Muller) incompatibilities, affects α and β, but the effect of selection on α is often more pronounced, particularly if dispersal from parental populations is limited [20,52]. Underdominance and epistatic DMIs thus tend to cause extreme genomic cline parameters (but note the latter are lacking in our study system).

### Study design and predictions

Related work in the *T. cristinae* system demonstrated that multiple geographic and ecological factors each leave clear patterns in the genome [44](Additional file 1: Figure S1, Additional file 2: Figure S2, Additional file 3: Figure S3). One major result was that the number of outlier loci tended to increase with greater geographic separation between populations, partially due to stronger selection (i.e., more pronounced environmental differences) between more distantly separated populations.

Here, we extend this previous work in two major ways. First, we provide novel analyses testing whether local adaptation results in selection against foreign alleles, a process that can reduce introgression and promote the genetic differentiation that drives speciation [10]. The admixed populations that we estimate introgression in are geographically much closer to one allopatric population (LA) than to the other. If divergent selection in allopatry causes exceptional genetic differentiation and if local adaptation along environmental gradients selects against foreign alleles (i.e., in this case, against alleles with PRC ancestry), we predict a greater number of F_ST_ outlier loci should exhibit elevated LA ancestry than elevated PRC ancestry (i.e., an excess of highly positive α values, relative to highly negative ones). We stress that this analysis relies strictly on the departure of individual loci from the genome-wide hybrid index (i.e., α of individual loci), rather than on average hybrid index (h) itself.

Second, we test for correspondence between locus-specific differentiation between allopatric populations and locus-specific introgression in admixed populations. Here we focus on absolute (i.e., unsigned) levels of α because although loci with highly negative versus highly positive α values differ in their probability of LA versus PRC ancestry, both loci with highly negative and highly positive α values exhibit exceptional patterns of introgression in admixed populations. A wide range of outcomes is possible because reproductive isolation can evolve via selection or drift and might evolve via similar or different processes in allopatric versus admixed populations. We focus here on some likely possibilities.

First, some regions might exhibit strong divergence between the allopatric populations and high (absolute) α values in the admixed populations. These likely represent regions that truly differentiated between LA and PRC via divergent selection, and which contribute to reproductive isolation in admixed populations (and potentially between allopatric populations as well). Second, some regions will exhibit low divergence and low (absolute) α values. These likely represent neutrally evolving regions that do not readily differentiate and which do not contribute to reproductive isolation. Third, some regions might exhibit strong divergence in allopatry but low (absolute) α in the admixed populations. Such regions might not harbor genes under divergent selection or those reducing introgression, but have nonetheless differentiated strongly in allopatry due to genetic drift. Alternatively such regions could harbor genes experiencing divergent selection in allopatry that are unrelated to reproductive isolation in admixed populations. Possible mechanisms causing such discordance between α and F_ST_ are geographic variation in the genomic architecture of local adaptation or in local selection pressures. Both these types of variability among populations can cause high F_ST_ and α to occur in some geographic regions, but not in others. Such factors could also explain loci with high α but low F_ST_. We enumerate the frequency of these different outcomes and discuss the results with respect to understanding speciation.

## Methods

### Description of the SNP dataset

The data we analyzed come from six of the eight populations examined by [44](n = 19–21 individuals per population). Two populations were excluded because they reside on a different mountain from the others. A total of 38,304 SNPs were obtained from Illumina sequencing of highly multiplexed genomically reduced fragment libraries. This represents a subset of the loci examined by [44], created by removing low variant loci (minor allele frequency < 0.10). Low variant loci are uninformative about ancestry, and could artificially inflate the correlation between F_ST_ and genomic cline parameter estimates [31]. Description of sampling and sequencing protocols, sequence assembly, variant calling, F_ST_ estimation and methods for delimitation of ‘outlier loci’ with exceptionally high F_ST_ values are described in full detail in [44]. Notably, both the current study and [44] used the same Bayesian analytical framework which ensures that missing data will not lead to high F_ST_, because it fully accounts for uncertainty in genotypes, allele frequencies, and F_ST_ caused by missing data and low sequence coverage. Low sequence coverage will simply cause greater uncertainty in F_ST_ (i.e., wide credible intervals) and will mean that F_ST_ for the locus will be more similar to the prior expectation (i.e., genome-average). In other words, for a locus to be an outlier or have a high F_ST_ there must be sufficient data for that locus for the likelihood (based on the data for that locus) to overcome the prior (based on the genome-average F_ST_).

We obtained highly congruent results from the two admixed populations and thus present mainly results from HV in the main text. Full results for MR are provided in the additional materials.

### Genomic cline estimation

We implemented a Bayesian genomic cline model that incorporates uncertainty in genotypic state inherent in next-generation sequence data [43], but is otherwise identical to the model described by [52]. We estimated marginal posterior probability distributions for hybrid indexes and cline parameters (α and β) using MCMC. We ran five independent chains for 50,000 steps each and recorded samples from the posterior distribution every 20th step following a 30,000 step burn-in. We combined the output of the five chains after inspecting the MCMC output to assess convergence to the stationary distribution. Significance of individual α values was assessed using 95% credible intervals. We detected very little variation in β and no loci departed significantly from a β value of zero. We thus present below only results for α.

### Comparative analyses

The zones of admixture we studied were geographically closer to LA than to PRC. If local adaptation varies relatively continuously along environmental gradients (potentially resulting in isolation-by-distance) and thus selects against foreign PRC alleles in the admixture zones, we predict an excess of loci with elevated LA ancestry (i.e., those with α > 0), relative to those with elevated PRC ancestry (i.e., those with α < 0). We thus tested whether high-F_ST_ outlier loci between PRC and LA were more likely to have elevated LA ancestry (α > 0) than expected by chance in admixed populations. The genomic cline parameter α represents a deviation from the ancestry probability predicted solely from hybrid index and is constrained to sum to zero. Thus, we used the expectation that 50% of outlier loci should have α > 0 as a null hypothesis. We tested for a significant deviation from this expectation based on a binomial probability distribution with pLA = 0.5. Additionally, we obtained Bayesian posterior estimates of the probability that F_ST_ outlier loci had estimates of α > 0 by specifying a binomial likelihood for the number of F_ST_ outlier loci with α > 0 and an uninformative beta prior on pLA (i.e., pLA ~ beta[1, 1]). We also conducted these analyses with all 38,304 loci for comparison.

We used correlational analyses to test for relationships between locus-specific F_ST_ and locus specific absolute values of α. We used the empirical quantiles of the estimated F_ST_ and absolute α values to delimit different combinations of high and low values for these two parameters (e.g., as discussed in the Study Design section). For F_ST_, we used the 95th quantile to determine loci with ‘low F_ST_’ (i.e., ~95% of loci get classified as 'low F_ST_'). For α, we are interested in extreme negative and positive values. Therefore we used the 2.5th and 97.5th empirical quantiles (i.e., ~95% of loci are classified as 'low α').

## Results

### Genomic introgression

**Figure 2:**
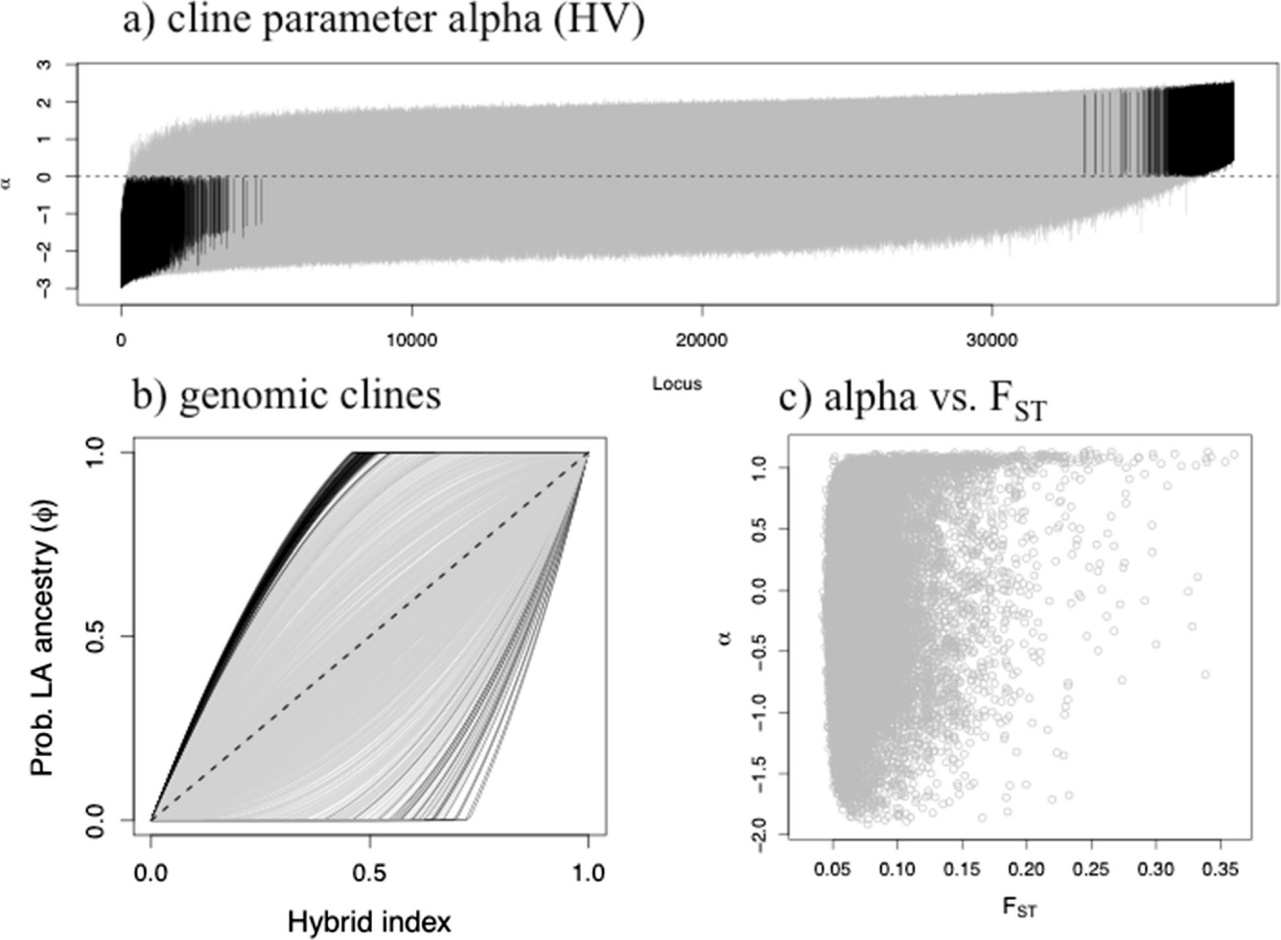
**Results of genomic clines analyses for the population pair HV (results for MR were similar; see Additional file****4****: Figure S4).a**) 95% credible intervals for genomic cline parameter α. Loci are sorted by the point estimate of α and 95% CI's that do not include zero are shown in black (i.e., introgression for these loci is significantly different than the genome-wide average). There are over 30,000 lines, thus individual 95% CI’s are difficult to see. **b**) Genomic clines for 1000 representative loci. Black lines denote clines with α values whose CI do not include zero. Grey lines denote loci whose α values had CI that did include zero. **c**) The correlation between F_ST_ and α. See text and Tables 1, 2 for statistics.

**Figure 3:**
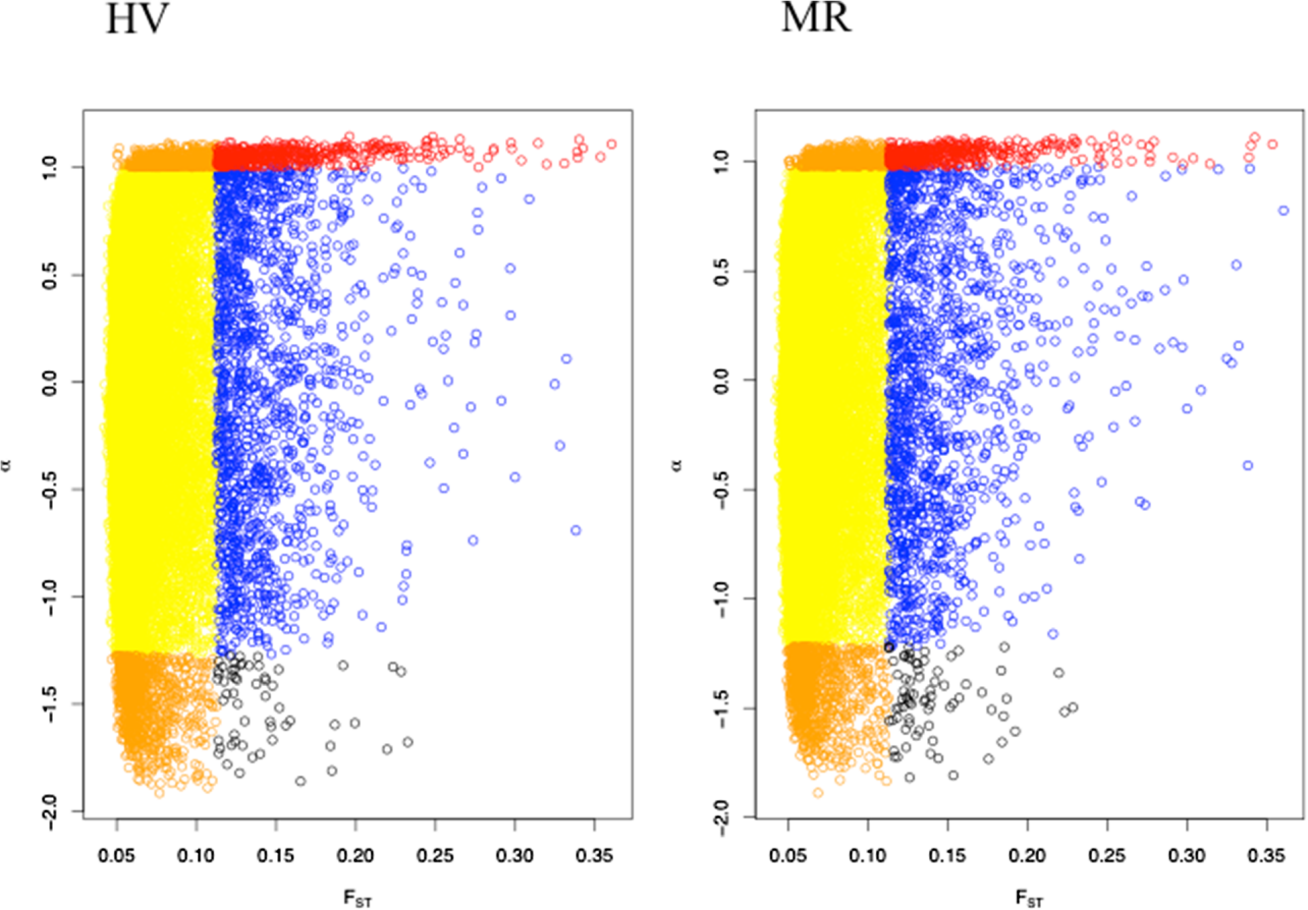
**Different combinations of locus-specific F**_**ST**_**and locus-specific α values.** Yellow denotes loci with both low F_ST_ and low absolute α values (i.e., putatively neutral, undifferentiated loci). Red denotes loci with both high F_ST_ and highly positive α values. Orange denotes loci with low F_ST_ but highly negative and highly positive' α values. Blue denotes loci with high F_ST_ but low α values. Black denotes loci with high F_ST_ and highly negative α values. ‘Low’ and ‘high’ values are delimited using the quantiles of the empirical distribution. See text for details.

**Figure 4:**
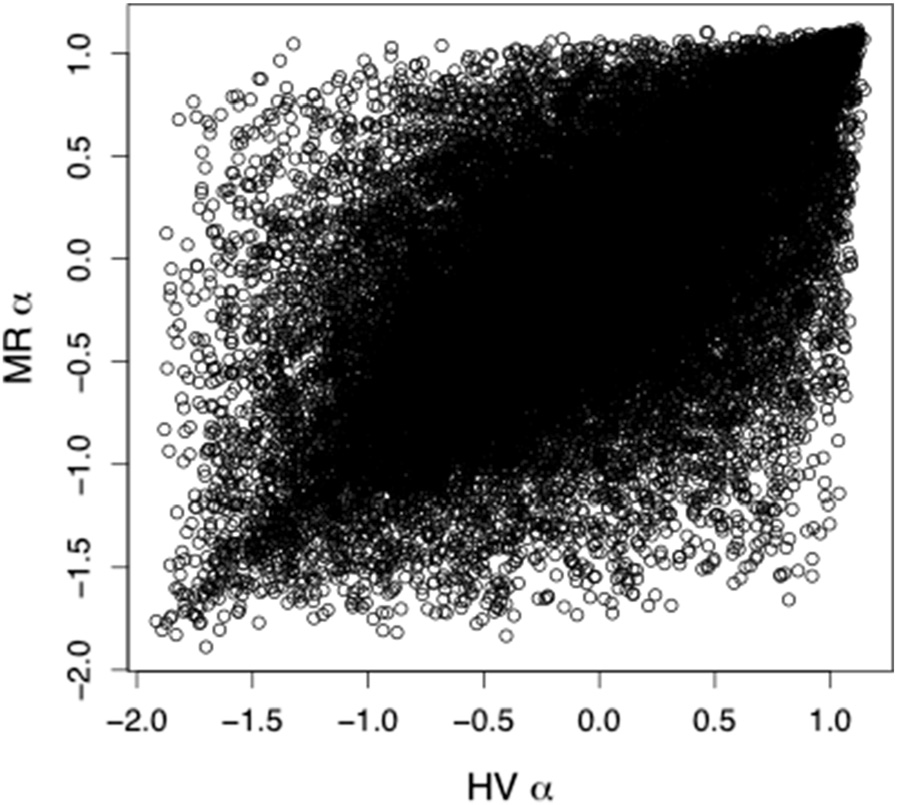
**The relationship between α for MR versus HV.** There was a strong (r = 0.64) relationship, but many loci that had high α values in one admixed population had low α values in the other (and vice-versa).

We observed substantial variation in genomic introgression (Figures 2, 3 and 4; Additional file 4: Figure S4). In HV, 1717 loci out of 38,304 had α values significantly greater than 0 (~4.5%) and 1056 loci had α values significantly less than 0 (~2.8%). Similarly, for MR 1517 loci had α significantly greater than 0 (~4.0%) and 925 had α significantly less than zero (~2.4%). The relationship between locus-specific α values from the two different admixed populations was substantial (r = 0.64). Nonetheless, many loci had different α values between the two admixed populations, indicative of geographically variable genetic drift, genetic architecture, or selective regimes (Figure 4). We detected little variation in hybrid index within each admixed population. Mean hybrid indices across loci were 0.72 (s.d. 0.01) and 0.71 (s.d. 0.01) for HV and MR, respectively.

### Directionality of α values

The fact that more loci with α values significantly different from zero were highly positive than highly negative provides the first indication that there was selection against foreign alleles in admixed populations. We conducted more explicit analyses examining this trend. We found that for high-F_ST_ outlier loci from the allopatric population pair there was a consistent and significant excess of highly positive α values relative to highly negative α values in the admixed populations (Table 1 for detailed results and statistics). This means that there were many outlier loci with elevated LA ancestry, and more so than loci with elevated PRC ancestry.

**Table 1:** Tests for whether α values tend to be greater than zero (i.e., excess of LA ancestry; p = probability; CI = confidence interval)

**Class of loci**	**Number of loci with α > 0**	**Number of loci with α < 0**	**Binomial probability**	**Bayesian posterior estimates of p_α >0 with 95% CI**
HV				
outliers	142	40	p = 1.4e-14	0.778 (0.714–0.834)
extreme outliers	17	4	p = 0.007	0.791 (0.597–0.922)
all loci	17328	20922	p = 2.2e-16	0.454 (0.449–0.459)
MR				
outliers	145	37	p = 1.6e-16	0.795 (0.732–0.849)
extreme outliers	18	3	p = 0.0015	0.836 (0.651–0.948)
all loci	17253	21051	p = 2.2e-16	0.450 (0.445–0.455)

Outliers are designated at the 95% level and extreme outliers at the 99% level. See text and [44] for details.

See text for details.

### Relationship between (absolute) α and F_ST_

Here we focus on absolute levels of α because although loci with highly negative versus highly positive α values differ in their probability of LA versus PRC ancestry, both loci with highly negative and highly positive α values exhibit exceptional patterns of introgression in admixed populations. We detected a highly significant positive correlation between F_ST_ and absolute values of α (HV: r = 0.24, p < 2.2e-16; MR: r = 0.24, p < 2.2e-16; Figure 3), indicative of relationships between genetic differentiation, divergent selection, and reproductive isolation. However, many loci departed from this general relationship, and sometimes strongly so. We thus enumerated the frequency of different combinations of high and low F_ST_ and high and low (absolute) α values (Table 2). At the 95% quantile level, 1.3% of loci exhibited both highly positive (absolute) α and high F_ST_ values, as expected for regions differentiating between allopatric populations via divergent selection that also affect reproductive isolation in admixed populations. A substantial proportion of loci exhibited low (absolute) α despite high F_ST_ (3.7%).

**Table 2:** **Numbers and percentages (in parentheses) of SNP loci falling into the four possible different combinations of (absolute) α and F**_**ST**_**values (high and low delimited at the 95% quantile level)**

**Population**	**Quantile**	**High α ; high F**_**ST**_	**Low α ; low F**_**ST**_	**High α ; low F**_**ST**_	**Low α ; high F**_**ST**_
HV	95	499 (1.3%)	34971 (91.3%)	1417 (3.7%)	1417 (3.7%)
MR	95	503 (1.3%)	34976 (91.3%)	1414 (3.7%)	1413 (3.7%)

See text for further details.

## Discussion and conclusion

When populations diverge in the face of gene flow, exceptionally differentiated genomic regions are often assumed to harbor genes undergoing reduced introgression whereas, in contrast, the remainder of the genome is homogenized by high introgression [1,5,8]. However, a number of factors other than low introgression might affect differentiation [29,55], leading to regions that are highly differentiated but relatively incidental to the speciation process [32,33]. Thus, tests of the relationship between genetic divergence, selection, and introgression are required.

Here we provided such a test and found some correspondence between locus-specific divergence and locus-specific introgression. Specifically, we detected accentuated ancestry from either allopatric population for highly differentiated loci, consistent with such highly divergent regions contributing to reproductive isolation in the admixed populations. There are reasons to expect such correspondence in the system examined; population sizes are large, introgression in admixed populations is known, admixed populations are relatively old, and gene flow from allopatric populations into admixed populations is low [44]. These are the conditions under which variation in introgression will more closely reflect locus-specific contributions to reproductive isolation, resulting in correspondence between locus-specific divergence and locus-specific introgression [52].

The observed correspondence between divergence and introgression is consistent with previous work in other systems documenting genomic islands of divergence [1,23-26,56] and with studies demonstrating that phenotypic traits contributing to divergent adaptation and reproductive isolation map to regions of the genome that are highly divergent between natural populations [38,57,58]. However, correspondence between divergence and genomic introgression in our study was only partial, and we discuss the implications of this below. Our results are consistent with a previous study in a butterfly that pioneered the approach we used here, and documented similar results [43]. Nonetheless, the current study extends previous work by explicitly considering the implications of different combinations of divergence and introgression.

### Local adaptation and selection against foreign alleles

We documented an excess of high-F_ST_ outlier loci with positive α values in admixed populations; that is, with accentuated LA ancestry. This result is suggestive of local adaptation to the environmental conditions around LA, which might be expected given that LA is more proximate to the admixture zones than is PRC. Most previous work on this system focused on the effects of divergent host adaptation [46]. However, a recent study suggests that adaptation in this system is multifaceted, and could also include climatic components and adaptation along continuous environmental gradients [44]. Thus, future work in this system could test the factors resulting in excess LA ancestry. The current results suggest that applying a genomic clines approach to a wider range of populations might be useful for understanding adaptation in the *Timema* system more generally. In particular, it would be interesting to estimate genomic introgression in admixed populations that are near PRC (which do exist in nature). The prediction would be an excess of loci with PRC ancestry, and this should occur for loci that had excess LA ancestry in the current study (and such contrasting patterns in admixture zones more proximate to LA versus PRC would be unlikely to arise due to the spread of universally favored variants).

Finally, we note that we did observe some loci that were strongly divergent between allopatric populations and had highly negative (rather than positive) α values. Although many selective pressures in the admixed populations studied here might mirror the conditions in LA, some might mirror those in PRC, particularly if selection is highly multifarious. Thus, these loci with highly negative α values could represent loci responding to conditions that mirror those in PRC (or possibly some subset of universally favored variants). Again, further studies of genomic introgression in admixed populations near PRC could test this prediction.

### Enumeration of correspondence between divergence and introgression

Our results show a general association between F_ST_ and α. However, many loci depart from this relationship, leading to the different outcomes discussed previously. If F_ST_ and α were completely independent, we would expect the frequency of the four following outcomes to be as follows (at the 95^th^ quantile level): high α, high F_ST_ = 0.05 * 0.05 = 0.25%; low α, low F_ST_ = 0.95 * 0.95 = 90.25%; high α; low F_ST_ = 0.05 * 0.95 = 4.75%; low α; high F_ST_ = 0.95 * 0.05 = 4.75%, etc. Instead, we observed an excess of loci falling into the first two categories, and a slight deficiency of loci falling into the latter two. This is exactly what is expected given the correlation between F_ST_ and α (i.e., given the non-independence of these two factors).

Some discordance between F_ST_ and α is expected in *T. cristinae*. For example, allopatric populations undergo little or no gene flow [44,47,50]. Thus, strong differentiation of regions that do not affect reproductive isolation in admixed populations can occur between allopatric populations via drift. Additionally, host-associated populations of *T. cristinae* exist in a mosaic patchwork and there is evidence that divergence in host use arose multiple times [46,59]. Thus, it is possible that geographic variation in genetic architecture amongst independently founded and evolving sets of populations contributes to departures from the association between F_ST_ and α. In summary, our results are consistent with some regions of strong differentiation harboring genes subject to divergent selection, but also often representing different outcomes such as divergence by drift or driven by geographic variation in genetic architecture or local selective regimes.

The findings shed insight into the theory of ‘genomic islands of speciation’ [23-26]. In this metaphor, ‘sea level’ represents the upper level of genetic differentiation possible via neutral processes. Most of the genome is homogenized by gene flow and exhibits differentiation that falls below sea level. A few regions of strong differentiation rise above sea level. These ‘genomic islands of speciation’ are comprised of outlier loci having genetic differentiation that exceeds background neutral expectations and may harbor genes affecting reproductive isolation [1,25,57]. However, our results suggest that highly divergent regions between populations undergoing little or no introgression might sometimes represent so-called ‘incidental islands’ that do not harbor genes affecting reproductive isolation [31,33], but that have nonetheless differentiated strongly via genetic drift. Further studies of the relationship between genetic divergence and reproductive isolation are warranted. If combined with information on the genomic position of the loci examined such work will allow explicit tests of the size, distribution, and number of ‘speciation’ versus ‘incidental’ islands.

### Caveats and considerations

There are three main caveats that should be taken into account when interpreting our results. First, loci that are more differentiated among populations carry more information about ancestry, and this could affect estimates of α. Thus, variation in the ancestry-information content among loci can generate a relationship between F_ST_ and absolute values of α, even in the absence of selection [43]. Previous work using computer simulations found that variation in ancestry content could generate correlations between F_ST_ and absolute values of α, but only for loci with low F_ST_ values [43]. For loci with high F_ST_ values (e.g., > 0.08), the relationship between F_ST_ and α in simulated datasets was erratic and not consistently positive.

Here, we estimated correlation coefficients for the association between F_ST_ and α in our data for loci with varying minimum F_ST_ values (from 0.02 to 0.18, in increments of 0.02). This was done for each admixed population separately. In the data, correlations between F_ST_ and α decrease within increasing F_ST_, particularly for loci with F_ST_ > 0.08 (Additional file 5: Figure S5). However, the correlation for HV remained consistently positive across the full range of F_ST_ values, unlike simulated data sets. This was not true of the correlation for MR, which hovers around zero. This result suggests that F_ST_ and α are more strongly related in HV than in MR, at least for moderately to highly differentiated loci. Finally, we stress that although simulated data can produce a correlation between F_ST_ and α for low F_ST_ loci, this does not mean that the empirical correlations observed here for loci with low F_ST_ are not real, but rather that they should be interpreted with caution. Finally, the correlation with higher F_ST_ values, particularly for HV, is unlikely to be due to variation in ancestry-information content among loci.

Second, the designation of allopatric populations used in the current study is imperfect because the geographic location of admixed populations makes it possible that they are more genetically distinct from one of the allopatric populations (i.e., PRC) than the allopatric populations are from each other (Additional file 1: Figure S1). However, past work does indicate strong divergence between the allopatric populations LA and PRC, on the order of that or greater than that observed between the admixed populations and PRC [44,46,59,60]. Moreover, divergence among the populations examined is not recent (generally on the order of hundreds of thousands of generations or more) [44,46,59], and thus it is unlikely the admixed populations arose recently from only one of the allopatric populations. Thus, our general and qualitative conclusions are likely to hold, but future work could examine the quantitative effects of using different allopatric populations.

Third, the excess of loci with highly positive, rather than highly negative, α values could arise if LA was exporting more universally beneficial mutations than PRC. This could occur, for example, if LA was a much larger population and because LA is closer to the admixed populations than PRC. A number of observations argue against the ‘biased mutation’ scenario. First, population sizes of LA and PRC are comparable (if anything, LA is smaller), as estimated from the size of the host-plant patch each population occupies (which is highly correlated with insect population size in this species) [50] and as estimated from coalescent-based estimates of effective population size from genomic data [44]. For example, host plant patch sizes for LA and PRC are 35,857 m^2^ and 68,181 m^2^, respectively. Finally, excess genome-wide LA ancestry within admixed populations, due to the closer proximity of LA to the admixed populations, also cannot explain our results because our analyses rely strictly on the departure of individual loci from genome-wide hybrid index, rather than on average hybrid index itself. Thus, local adaptation to conditions surrounding LA seems a more plausible explanation than biased mutation for an excess of loci with highly positive α values.

### Future directions

The approach and results presented here provide a test of the idea that genomic regions of strong divergence can harbor genes that are important for speciation. However, a number of further lines of evidence could be used to strengthen this conclusion, and to refine the results. For example, it will be of interest to learn the genomic distribution of the loci examined and to test which regions are linked to phenotypic traits involved in divergent adaptation. Such mapping studies are currently underway. Additionally, several field experiments designed to measure selection at the genomic level have now been implemented in *T. cristinae*. These experimental results can then be coupled with the genomic clines analyses presented here and functional genomic studies to gain a more complete picture of the associations among genetic divergence, selection, reproductive isolation, and speciation.

Finally, the current study examined populations that are at a particular point in the speciation process, and an early one where reproductive isolation is far from complete [46]. The results presented here provide only a ‘snapshot’ of the often-extended process of speciation. Future work could extend the approaches considered here to taxon-pairs of *Timema* spanning a wider range of divergence (e.g., hybridizing species pairs) in an attempt to reconstruct how the relationship between genetic divergence and reproductive isolation might change as the continuous process of speciation unfolds.

## Competing interest

The authors declare no competing interests.

## Authors' contributions

PN, TP, JF, and ZG conceived the project. PN, TP, and ZG conducted the analyses. PN, TP, JF, and ZG wrote the manuscript. All authors read and approved the final manuscript.

## Supplementary Material

Additional file 1 **Figure S1.** TMap of the eight study populations examined by Nosil et al. [44]. The current study examines all populations except R12A and R12C. See text for details. Click here for file

Additional file 2 **Figure S2.** The relationship between the number of outlier loci (on log10 scale) and the geographic distance between populations (on log10 scale), for geographically separated and geographically adjacent population pairs (filled and unfilled circles, respectively). Both the effects of geographic distance itself, and of geographic arrangement (separated versus adjacent) were statistically significant. The thick arrow labels the point at which zero gene flow between population pairs was achieved, where gene flow was estimated from genomic data using Approximate Bayesian Computation. Also shown is a picture of the study organism. Modified from Nosil et al. [44]. Click here for file

Additional file 3 **Figure S3.** The distribution of F_ST_ values under different degrees of geographic separation. The top panel shows the distribution of F_ST_ values across individual loci. The bottom panel presents a barplot of the distribution of point estimates for logit(F_ST_) across the genome. The dashed black line is the genome-wide distribution of logit(F_ST_) (i.e., the Gaussian normal hierarchical prior for locus-specific logit(F_ST_)). The vertical red line in each pane denotes the 95th quantile of the genome-wide distribution, which was used to delimit high F_ST_ outliers. In all instances, some outlier loci were detected but the F_ST_ distribution tended to be the most ‘L-shaped’ for geographically-adjacent pairs and became less ‘L-shaped’ with increasing geographic separation of populations. Modified from Nosil et al. [44]. Click here for file

Additional file 4 **Figure S4.** Results of genomic clines analyses for the population pair MR. a) the 95% credible intervals for genomic cline parameters α. Loci are sorted by the point estimate of α and 95% CI's that do not include zero are shown in black (i.e., introgression for these loci is significantly different than the genome-wide average). There are over 30,000 lines, thus individual 95% CI’s are difficult to see. b) Genomic clines for 1000 representative loci. Black lines denote clines with α values whose CI do not include zero. Grey lines denote loci whose α values had CI that did include zero. c) The correlation between F_ST_ and α. See text and Tables 1, and 2 for statistics. Click here for file

Additional file 5 **Figure S5.** The correlation coefficient for the relationship between locus-specific F_ST_and locus-specific absolute α values (y-axis) for loci with F_ST_ values above certain thresholds (x-axis). Open circles are HV. Closed circles are MR. Numbers of loci that had F_ST_ values above each threshold are as follows (0.02: 38304; 0.04: 38304; 0.06: 21937; 0.08: 5680; 0.10: 2922; 0.12: 1548; 0.14: 858; 0.16: 477; 0.18: 282). Click here for file
