# Supplementary material for: Do highly divergent loci reside in genomic regions affecting reproductive isolation? A test using next-generation sequence data in Timema stick insects
Source: BMC Evol Biol. 2012 Aug 31;12:164. doi: 10.1186/1471-2148-12-164 (PMC3502483; doi:10.1186/1471-2148-12-164)
Supplement: Additional file 1 — Figure S1. TMap of the eight study populations examined by Nosil et al. [44]. The current study examines all populations except R12A and R12C. See text for details. [file 1471-2148-12-164-S1.docx]

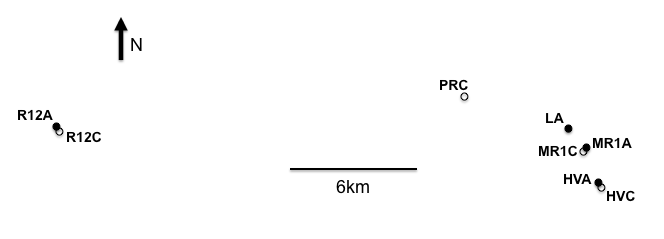
a

Additional file: Figure S1. Map of the eight study populations examined by Nosil et al. [44]. The current study examines all populations except R12A and R12C. See text for details.
