# Supplementary material for: Do highly divergent loci reside in genomic regions affecting reproductive isolation? A test using next-generation sequence data in Timema stick insects
Source: BMC Evol Biol. 2012 Aug 31;12:164. doi: 10.1186/1471-2148-12-164 (PMC3502483; doi:10.1186/1471-2148-12-164)
Supplement: Additional file 2 — Figure S2. The relationship between the number of outlier loci (on log10 scale) and the geographic distance between populations (on log10 scale), for geographically separated and geographically adjacent population pairs (filled and unfilled circles, respectively). Both the effects of geographic distance itself, and of geographic arrangement (separated versus adjacent) were statistically significant. The thick arrow labels the point at which zero gene flow between population pairs was achieved, where gene flow was estimated from genomic data using Approximate Bayesian Computation. Also shown is a picture of the study organism. Modified from Nosil et al. [44]. [file 1471-2148-12-164-S2.docx]

Additional file 2: Figure S2. The relationship between the number of outlier loci (on log10 scale) and the geographic distance between populations (on log10 scale), for geographically separated and geographically adjacent population pairs (filled and unfilled circles, respectively). Both the effects of geographic distance itself, and of geographic arrangement (separated versus adjacent) were statistically significant. The thick arrow labels the point at which zero gene flow between population pairs was achieved, where gene flow was estimated from genomic data using Approximate Bayesian Computation. Also shown is a picture of the study organism. Modified from Nosil et al. [44].
