# Supplementary material for: Do highly divergent loci reside in genomic regions affecting reproductive isolation? A test using next-generation sequence data in Timema stick insects
Source: BMC Evol Biol. 2012 Aug 31;12:164. doi: 10.1186/1471-2148-12-164 (PMC3502483; doi:10.1186/1471-2148-12-164)
Supplement: Additional file 3 — Figure S3. The distribution of FST values under different degrees of geographic separation. The top panel shows the distribution of FST values across individual loci. The bottom panel presents a barplot of the distribution of point estimates for logit(FST) across the genome. The dashed black line is the genome-wide distribution of logit(FST) (i.e., the Gaussian normal hierarchical prior for locus-specific logit(FST)). The vertical red line in each pane denotes the 95th quantile of the genome-wide distribution, which was used to delimit high FST outliers. In all instances, some outlier loci were detected but the FST distribution tended to be the most ‘L-shaped’ for geographically-adjacent pairs and became less ‘L-shaped’ with increasing geographic separation of populations. Modified from Nosil et al. [44]. [file 1471-2148-12-164-S3.docx]

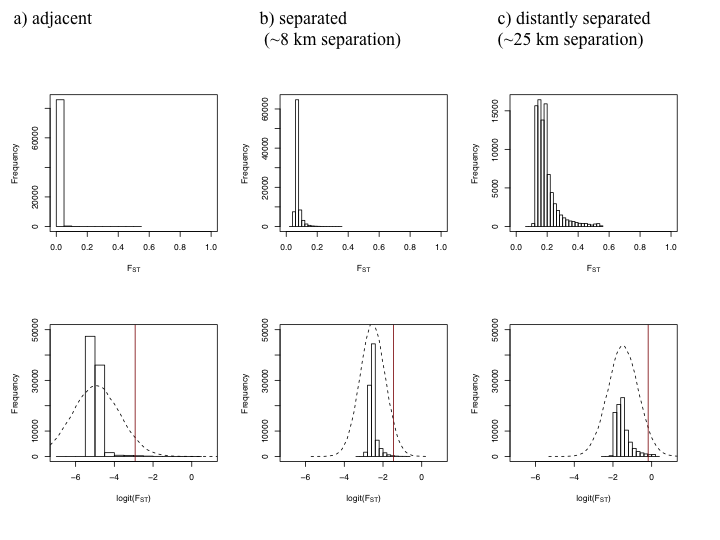


Additional file 3: Figure S3. The distribution of F_ST_ values under different degrees of geographic separation. The top panel shows the distribution of F_ST_ values across individual loci. The bottom panel presents a barplot of the distribution of point estimates for logit(F_ST_) across the genome. The dashed black line is the genome-wide distribution of logit(F_ST_) (i.e., the Gaussian normal hierarchical prior for locus-specific logit(F_ST_)). The vertical red line in each pane denotes the 95th quantile of the genome-wide distribution, which was used to delimit high F_ST_ outliers. In all instances, some outlier loci were detected but the F_ST_ distribution tended to be the most ‘L-shaped’ for geographically-adjacent pairs and became less ‘L-shaped’ with increasing geographic separation of populations. Modified from Nosil et al. [44].
