# Supplementary material for: Do highly divergent loci reside in genomic regions affecting reproductive isolation? A test using next-generation sequence data in Timema stick insects
Source: BMC Evol Biol. 2012 Aug 31;12:164. doi: 10.1186/1471-2148-12-164 (PMC3502483; doi:10.1186/1471-2148-12-164)
Supplement: Additional file 4 — Figure S4. Results of genomic clines analyses for the population pair MR. a) the 95% credible intervals for genomic cline parameters α. Loci are sorted by the point estimate of α and 95% CI's that do not include zero are shown in black (i.e., introgression for these loci is significantly different than the genome-wide average). There are over 30,000 lines, thus individual 95% CI’s are difficult to see. b) Genomic clines for 1000 representative loci. Black lines denote clines with α values whose CI do not include zero. Grey lines denote loci whose α values had CI that did include zero. c) The correlation between FST and α. See text and Tables 1, and 2 for statistics. [file 1471-2148-12-164-S4.docx]

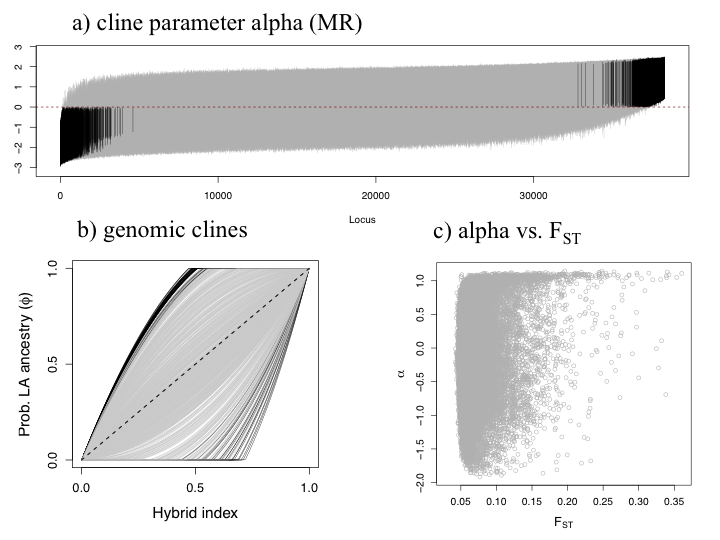


Additional file 4: Figure S4. Results of genomic clines analyses for the population pair MR. a) the 95% credible intervals for genomic cline parameters α. Loci are sorted by the point estimate of α and 95% CI's that do not include zero are shown in black (i.e., introgression for these loci is significantly different than the genome-wide average). There are over 30,000 lines, thus individual 95% CI's are a difficult to see. b) Genomic clines for 1000 representative loci. Black lines denote clines with α values whose CI do not include zero. Grey lines denote loci whose α values had CI that did include zero. c) The correlation between F_ST_ and α. See text and Tables 1, 2 for statistics.
