# Supplementary material for: Do highly divergent loci reside in genomic regions affecting reproductive isolation? A test using next-generation sequence data in Timema stick insects
Source: BMC Evol Biol. 2012 Aug 31;12:164. doi: 10.1186/1471-2148-12-164 (PMC3502483; doi:10.1186/1471-2148-12-164)
Supplement: Additional file 5 — Figure S5. The correlation coefficient for the relationship between locus-specific FSTand locus-specific absolute α values (y-axis) for loci with FST values above certain thresholds (x-axis). Open circles are HV. Closed circles are MR. Numbers of loci that had FST values above each threshold are as follows (0.02: 38304; 0.04: 38304; 0.06: 21937; 0.08: 5680; 0.10: 2922; 0.12: 1548; 0.14: 858; 0.16: 477; 0.18: 282). [file 1471-2148-12-164-S5.docx]

Additional file 5: Figure S5. The correlation coefficient for the relationship between locus-specific F_ST_ and locus-specific absolute α values (y-axis) for loci with F_ST_ values above certain thresholds (x-axis). Open circles are HV. Closed circles are MR. Numbers of loci that had F_ST_ values above each threshold are as follows (0.02: 38304; 0.04: 38304; 0.06: 21937; 0.08: 5680; 0.10: 2922; 0.12: 1548; 0.14: 858; 0.16: 477; 0.18: 282).

|  |
| --- |

|  |
| --- |

|  |
| --- |
